# Do confidence ratings prime confidence?

**DOI:** 10.3758/s13423-018-1553-3

**Published:** 2019-01-10

**Authors:** Kit S. Double, Damian P. Birney

**Affiliations:** 1 Department of Education, University of Oxford, Oxford, UK; 2 School of Psychology, University of Sydney, Sydney, NSW 2000 Australia

**Keywords:** Reactivity, Confidence ratings, Priming, Metacognition

## Abstract

Confidence ratings (CR) are one of the most frequently used measures in psychological research. However, recent evidence has suggested that eliciting CR from participants may result in changes to cognitive performance, so called *reactivity*. Here, we examine whether reactivity to CR can be better explained by added task-relevant introspection, or, alternatively, the unintentional priming of confidence-related beliefs. First, we compare participants’ performance in a group making CR with a group making a task-irrelevant control rating, and a second group who made the same task-irrelevant rating, but with the word ‘confident’ included in the rating’s wording. The results suggest that reactivity is driven by the presentation of the word ‘confident’, and reactivity does not require task-relevant introspection. Additionally, we show that rephrasing CR to remove the word ‘confident’ neutralises reactivity. This suggests that reactivity may represent a significant problem for researchers using CR, but rephrasing CR may remedy these concerns in relatively simple fashion.

Confidence ratings are commonly used within metacognitive research to assess the effectiveness of metacognitive monitoring (Fleming & Lau, 2014). Confidence ratings are generally elicited in an ‘online’ fashion, after each item on a task. Overall, confidence ratings are strongly related to accuracy in a range of domains, including general knowledge tests (Perfect, Watson, & Wagstaff, 1993), perceptual decisions (Fleming, Weil, Nagy, Dolan, & Rees, 2010), and reasoning tasks (Stankov, 2000). There are, however, significant individual differences in metacognitive ability as captured by confidence ratings (Fleming et al., 2010). While, confidence ratings have been utilised to provide valuable insights into metacognitive processes, they have typically been elicited with little consideration as to their effect on the underlying cognitive process. Here, we directly examine the effect of performing confidence ratings on reasoning performance, while isolating possible mechanisms responsible for such effects.

*Reactivity* occurs when performing a self-report measure causes a change in performance on a task. Reactivity can be positive, when performance improves, or negative, when performance declines. Methods for assessing metacognition may be particularly prone to reactivity, as they often involve collecting ‘online’ self-report measures while a participant performs a cognitive task. In theory, reactivity should occur when, and only when, a self-report measure elicits information that would not have otherwise been attended to (Ericsson & Simon, 1993). This claim was supported by a meta-analysis of 94 studies conducted by Fox, Ericsson and best (2011) that found think-aloud procedures that do not demand additional information from a subject (i.e. they require only that the subjects vocalises their current cognitions) were not reactive, whereas protocols that directed subjects for additional information, such as to provide explanations for their thought processes, displayed positive reactivity. Similarly, another meta-analysis of different metacognitive rating, judgements of learning, showed that in some circumstances, they, too, are reactive (Double, Birney, & Walker, 2018). Notably, the analysis showed that judgements of learning were only (positively) reactive when they were elicited from related, but not unrelated, word pairs, which suggests that task-characteristics can also influence reactivity.

Petrusic and Baranski (2003) provided one of the first examinations of reactivity to confidence ratings during a perceptual choice task. They found that confidence increased decision-making times, but did not significantly affect decision accuracy. However, as the authors noted, errors were higher when confidence ratings were elicited on 80% of the stimuli used, and the failure to reach significance was possibly a Type II error due to their small sample size (*N* = 28). A subsequent study by Birney, Beckmann, Beckmann, and Double (2017), in a sample of business managers, found that, compared with a no-rating control, participants who provided confidence ratings had impaired performance on Raven’s Progressive Matrices. Performance was particularly impaired on the difficult items of the task. However, Double and Birney (2018) recently showed that the direction of reactivity to confidence ratings depends on the preexisting self-confidence of participants (measured separately using a self-report measure). Participants with high preexisting confidence performed better on Raven’s Progressive Matrices, whereas performance was impaired when confidence ratings were collected from participants with low preexisting self-confidence, and these effects remained even after cognitive ability was controlled for. This led the authors to argue for the *cognizant confidence* hypothesis, which proposes that rather than providing a general benefit to cognitive performance through metacognitive reflection, confidence ratings prime preexisting confidence and thus have contrasting effects on high versus low confidence participants.

If confidence ratings do indeed differentially affect high and low confidence participants, it is not yet clear what it is about confidence ratings that leads to such effects. Two hypothesised mechanisms appear most likely to account for the effect. Firstly, it may be that the repeated presentation of the word ‘confident’ (as in ‘How confident are you that your last response was correct?’) primes participants’ preexisting self-confidence, which in turn affects their performance. Evidence suggests that goals and motivation can be unconsciously primed such that when one pays attention to a stimulus, the probability that the individual becomes consciously aware of it increases (Dijksterhuis & Aarts, 2010). We argue that the repetition of the word ‘confident’ makes participants more consciously aware of their subjective confidence, which in turn effects their performance, because self-confidence is an important determinant of performance (Bandura, 1993; Schunk, 1989; Zimmerman, 2000).

Alternatively, confidence ratings may result in self-confidence determined changes in reasoning performance because they represent an explicit demand to reflect on one’s performance after each decision. The introspection caused by this demand may have a dynamic effect on participants as they progress through the task and gain insights into the effectiveness (or not) of their reasoning strategies, which they are able to apply to later items. In support of this notion, there is some evidence that metacognitive interventions are effective at improving performance (Azevedo, 2005; Gagniere, Betrancourt, & Detienne, 2012). However, the effect of metacognitive introspection is likely to depend on self-confidence. While high self-confidence participants may benefit from the enhanced monitoring in the manner outlined, for low self-confidence participants it may trigger task-irrelevant processing, anxiety, and self-doubt. This, in turn, can have a negative impact on subsequent performance (Bouffard, Boisvert, Vezeau, & Larouche, 1995; Heslin, Latham, & VandeWalle, 2005; Zimmerman, 2000).

The current study investigates the mechanism(s) underlying reactivity to confidence ratings by assessing the effect of eliciting confidence ratings during Raven’s Progressive Matrices (RPM). In Experiment 1, we compare RPM performance of a group that performs the task with confidence ratings with two active control groups that make task-irrelevant ratings after each item. The task-irrelevant rating includes the word ‘confident’ in one of the groups, but not the other. Hence, we can determine whether the word 'confident' is in fact priming preexisting self-confidence, or if, instead, task-relevant introspection (i.e. reflection on one’s task performance) is necessary for reactivity to occur. To preempt the results, we find that priming appears to be driving reactivity effects, and thus, in Experiment 2, we examine the extent to which reactivity occurs to confidence ratings if they are rephrased to remove the word ‘confident’.

## Experiment 1

### Method

#### Participants and materials

A power analysis using G*Power was performed to determine the intended sample size. An effect size of *d =* .4 was utilised in the analysis, as effect sizes in this region have been found by previous studies (e.g. Double & Birney, 2017a). The analysis indicated a recommended sample of 111. We attempted to meet this recommendation, as well as recruit a number of extra participants in case some participants’ data needed to be excluded. One hundred twenty-five participants (62.4% female) were recruited using Amazon’s Mechanical Turk (*M*_age_ = 33.73 years, *SD* = 7.81 years). Participants for whom two or fewer correct responses were recorded were automatically discarded (*n* = 23). All participants completed RPM. After each RPM item, participants provided a particular rating depending on the condition to which they were randomly allocated. The confidence rating (CR) group (*n* = 34) had to provide *confidence* ratings; the priming (prime) group (*n* = 34) rated how *confident* two squares were identical in colour; and the task-irrelevant rating (control) group (*n* = 34), rated the *extent* to which two squares were the same colour. The same squares were used in the prime and control groups. All materials were administered using Qualtrics (Qualtrics, 2017).

##### Raven’s progressive matrices (RPM; Raven & Court, 1998)

Participants completed one of two computerised 12-item sets drawn from the Ravens’ Advanced Progressive Matrices Task (the complete set was counterbalanced across participants). Reduced versions of RPM have been shown to have concurrent validity and predictive power similar to the full version (Bors & Stokes, 1998). Items were ordered in the traditional fashion, which roughly corresponds to ascending difficulty.

##### Ratings

**Fig. 1:**
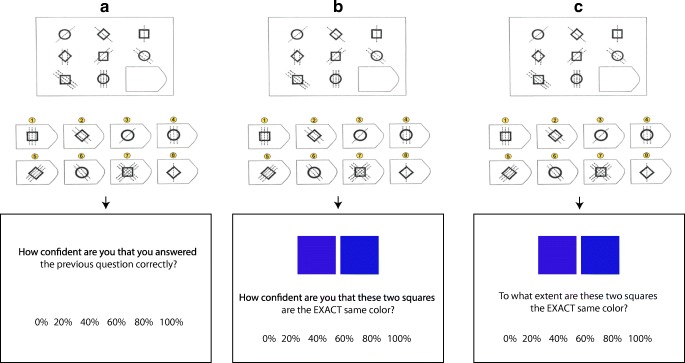
Experimental procedure for (**a**) the CR group, (**b**) the priming group, and (**c**) task-irrelevant rating group

All groups made their confidence rating on the same 6-point scale ranging from 0% to 100%. The confidence ratings were displayed after a participant’s response, and participants could neither view nor change their earlier question/answer when making the confidence rating. The CR group was asked, ‘How confident are you that you answered the previous item correctly?’ The prime and control groups were shown two coloured squares while making their rating. On half of the trials, the squares were identical in colour, while on the other half, they differed slightly in terms of colour. Both groups saw the same squares. Participants in the priming group were asked, ‘How confident are you that these two squares are the EXACT same colour?’ Participants in the control group were asked, ‘To what extent are these two squares the EXACT same colour?’ See Fig. 1 for an example of study materials.

##### Predicted cognitive ability (PCA)

In order to measure confidence proximally for the current task, we assessed participants’ predictions of their performance before completing the task. After reading the instructions for RPM and seeing two example items, participants were asked. ‘Before you begin, please predict your overall score on the test as a percentage.’ The example items shown were two easy problems that were both actual Raven’s items, but drawn from the standard set rather than the advanced (Items 27 and 40). Participants made their prediction on a continuous scale from 0 to 100.

### Results

All data analysis was performed using R (Version 3.4.1; R Core Team, 2017). Plots were produced with the ggplot2 package (Wickham, 2009). Descriptive statistics are available in Table 1. We utilised a linear regression model to examine the extent to which PCA moderated the effect of experimental group. The dummy coded group effect and PCA (mean centred) as well as their interaction were entered as predictors. The task-irrelevant rating (control group) was entered as the reference group. In addition, sex and age were entered as covariates in the model to control for demographic effects. Standardized betas are provided as a metric of effect size.

**Table 1:** Descriptive statistics for Experiment 1

Measure	*M* (*SD*)	2	4	4
1. Sex	1.37 (0.49)	.13	−.05	−0.20**
2. Age	34.23 (8.12)		.01	.07
3. PCA	71.26 (16.71)			.17
4. RPM	7.04 (2.46)			

PCA = predicted cognitive abilities; RPM = Raven’s Progressive Matrices. Sex was coded 1 = male, 0 = female. **p < .001

**Fig. 2:**
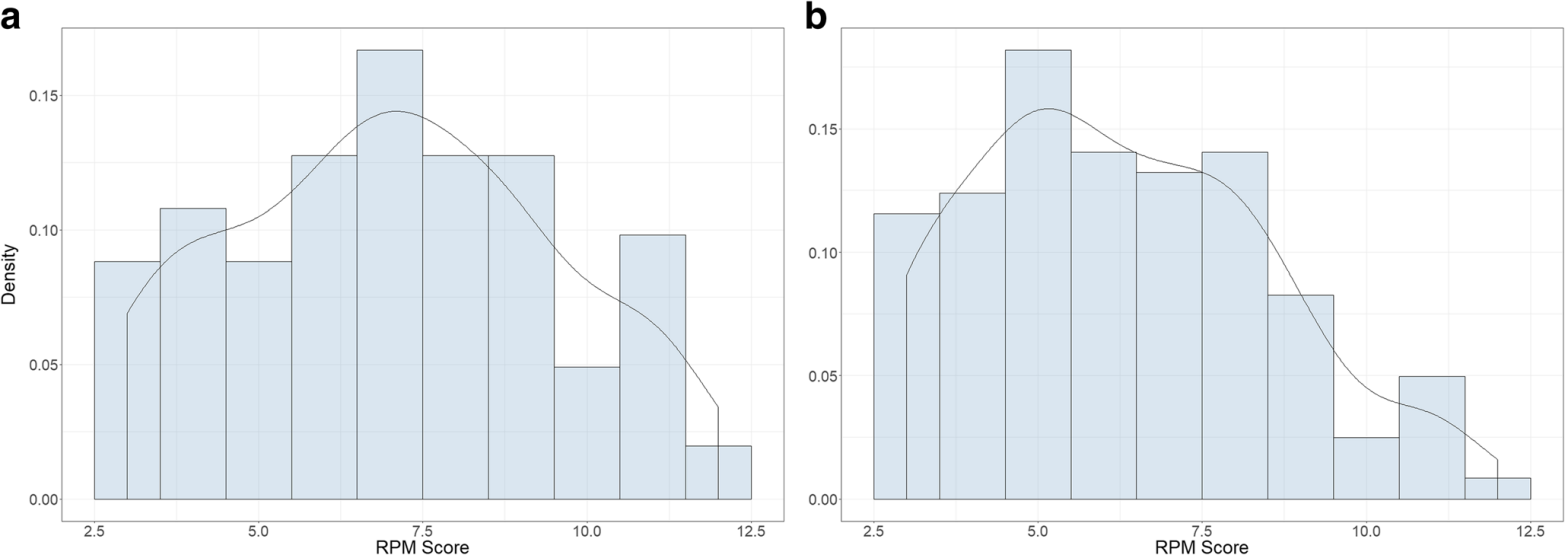
Density distributions of Raven’s Progressive Matrices (RPM) scores for (**a**) Experiment 1 and (**b**) Experiment 2

**Fig. 3:**
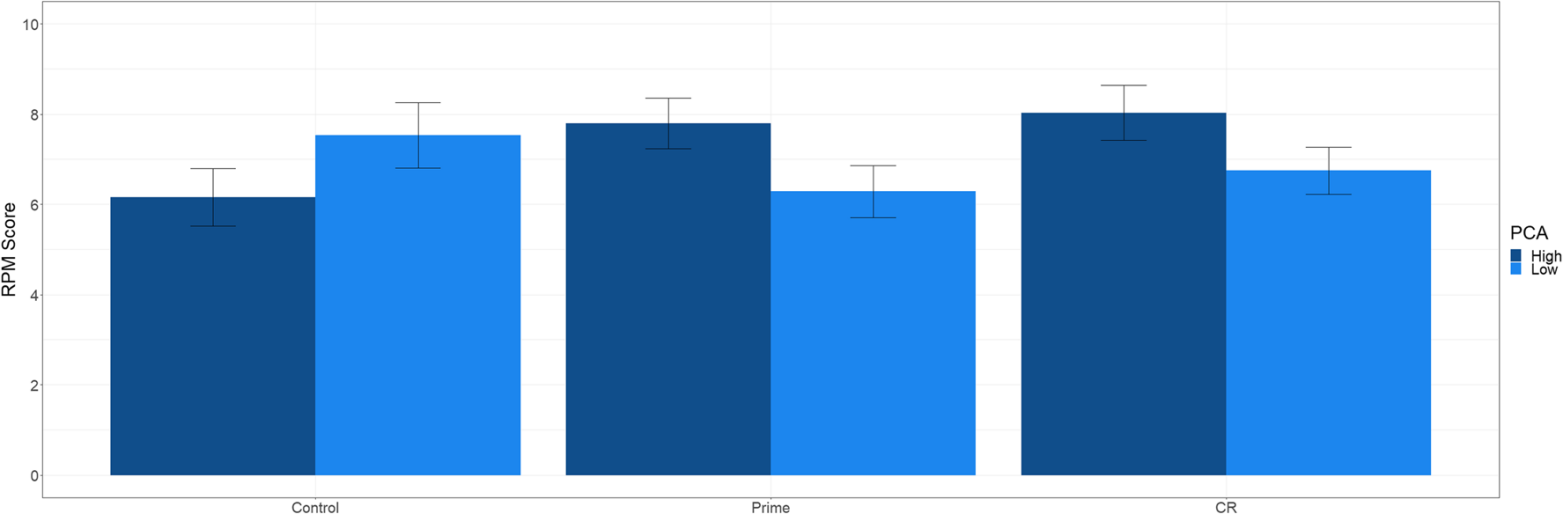
Raven’s Progressive Matrices (RPM) score (out of 12) as a function of experimental group and predicted cognitive abilities (PCA) for Experiment 1. Moderator values of one standard deviation above and below the mean were used for high and low PCA, respectively. Error bars represent +1 standard error of the mean

Density plots of RPM score are presented in Fig. 2a. The results suggest that there was no significant overall difference between the CR group (*M* = 7.06) and the control group (*M* = 6.88); β = 0.10, *t* = 0.91, *p* = .365. Similarly, there was no overall difference between the prime (*M* = 7.18) and the control group; β = .04, *t* = 0.33, *p* = .740. PCA was not a significant predictor of RPM score; β = −.28, *t* = −1.27, *p* = .209.^1^ This was qualified by a significant interaction between PCA and the CR versus control effect; β = .35, *t* = 2.00, *p* = .048. Similarly, there was a significant interaction between PCA and the prime versus control group effect; β = .36, *t* = 2.15, *p* = .034. Depicted in Fig. 3, high PCA individuals did substantially better in the CR and prime groups, compared with controls, whereas low PCA participants did marginally worse. For completeness, we reperformed the analysis using the CR condition as the reference group. The CR group did not differ significantly from the prime group, β = −.07, *t* = .57, *p* = .570. Furthermore, the prime versus CR group effect did not significantly interact with PCA, β = .03, *t* = .22, *p* = .828.

As a follow-up analysis we probed the moderation using using Hayes and Montoya’s (2017) method for testing multicategory interaction terms in linear regression. This method examines the pairwise comparisons between groups for different values of the moderator (i.e. PCA), while controlling for error inflation. We utilised the 25th, 50th, and 75th percentile as suggested by Hayes and Montoya (2017). The analysis was implemented using the PROCESS macro in SPSS (Hayes, 2017). There was a significant difference between the CR group and the control group for high PCA participants (*t* = 2.04, *p* = .044), whereas the difference between the prime group and the control group was just above conventional significance for high PCA participants (*t* = 1.88, *p* = .064). There were no significant group differences for moderate or low PCA participants (all *p*s > .10).

## Experiment 2

The results of Experiment 1 reconfirms previous studies that suggest that confidence ratings are reactive (Birney et al., 2017; Double & Birney, 2017a). Furthermore, the findings suggest that reactivity to confidence ratings is a consequence of repeatedly presenting the word ‘confident’ to participants. The relationship between PCA and performance was positive only in the CR and prime groups, suggesting that the relationship between PCA and performance was exaggerated whenever the word ‘confident’ was present in intertrial ratings. Based on this finding, in Experiment 2 we assess whether reactivity to confidence ratings can be reduced or even eliminated by rephrasing the confidence ratings to remove the word ‘confident’.

### Method

#### Participants and materials

Sample size was determined using the same method as Experiment 1, indicating a desired sample size of 111. Due to the larger than expected number of participants that had to be excluded in Experiment 1, we recruited a slightly larger number of participants to ensure that power was adequate in the final sample. One hundred and sixty-two participants (55.6% female) were recruited using Amazon’s Mechanical Turk (*M*_age_ = 35.85 years, *SD* = 11.48 years). Participants for whom two or fewer correct responses were recorded were automatically discarded (*n* = 41). All participants completed RPM in the same fashion described in Experiment 1. After each RPM item, participants provided a particular rating. The CR group (*n* = 40) again had to provide *confidence* ratings (‘How confident are you that you answered the previous item correctly?’). The likelihood group (*n* = 42) rated how likely it was that their previous answer was correct (‘How likely is it you that you answered the previous item correctly?’). Finally, the control group (*n* = 39) again rated the *extent* to which two squares were the same colour. PCA was assessed in the same manner as Experiment 1.

### Results

All data analysis was performed in the same fashion as Experiment 1. Descriptive statistics are available in Table 2. We utilised a linear regression model to examine the extent to which PCA moderated the effect of experimental group. The dummy coded group effect and PCA (mean centred) as well as their interaction were entered as predictors. The task-irrelevant rating (control group) was entered as the reference group. In addition, sex and age were entered as covariates in the model to control for demographic effects.

**Table 2:** Descriptive statistics for Experiment 2

Measure	*M* (*SD*)	2	3	4
1. Sex	1.49 (.5)	.12	−.24^**^	−.07
2. Age	36.55 (11.46)		−.09	.12
3. PCA	68.79 (20.63)			.22^*^
4. RPM	6.28 (2.25)			

PCA = predicted cognitive abilities; RPM = Raven’s Progressive Matrices. Sex was coded 1 = male, 0 = female. *p < .001

**Fig. 4.**
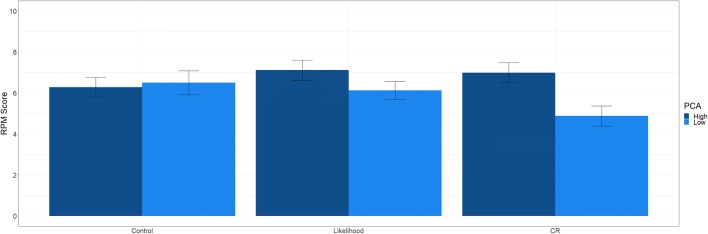
Raven’s Progressive Matrices (RPM) score (out of 12) as a function of experimental group and predicted cognitive abilities (PCA) for Experiment 2. Moderator values of one standard deviation above and below the mean were used for high and low PCA, respectively. Error bars represent +1 standard error of the mean

Density plots of RPM score are presented in Fig. 2b. The results suggest that there was no significant overall difference between the CR group (*M* = 5.98) and the control group (*M* = 6.33); β = −.10, *t* = 0.92, *p* = .361. Similarly, there was no overall difference between the likelihood (*M* = 6.52) and the control group; β = .17, *t* = 0.47, *p* = .641. PCA was not a significant predictor of RPM score; β = −.05, *t* = .28, *p* = .780. This was qualified by a significant interaction between PCA and the CR versus control effect; β = 0.30, *t* = 2.22, *p* = .029. As shown in Fig. 4, this effect was largely driven by impaired performance of low PCA participants in the CR group. There was no significant interaction between PCA and the likelihood versus control group effect; β = .17, *t* = 1.19, *p* = .237. For completeness we also reran the analysis with the CR group as the reference group. The results suggest that the CR group was not different to the likelihood group in terms of overall performance, β = .15, *t* = 1.41, *p* = .160. The difference between PCA and the CR versus likelihood was not significant; β = −.16, *t* = 1.19, *p* = .236.

As a follow-up analysis, we again probed the moderation using the PROCESS macro in SPSS. As with Experiment 1, we examined group differences for low, moderate, and high PCA participants (25th, 50th, and 75th percentiles). There was a significant difference between the CR group and the control group for low PCA participants (*t* = −2.09, *p* = .039), whereas the difference between the likelihood group and the control group was not significant (*t* = −.473, *p* = .637). There were no significant group differences for moderate or high PCA participants (all *p*s > .10). While this finding is different from Experiment 1, where the group differences were largely in low PCA participants, the pattern of results is similar between studies in that the effect of confidence on performance is exaggerated in conditions where the word ‘confident’ is presented to participants.

#### Metacognitive accuracy

As both the CR and likelihood groups provided task-relevant judgements of their performance, it was possible to examine BOTH the effect of judgement type on metacognitive accuracy and whether participants' metacognitive accuracy interacted with group effect. We operationalised metacognitive accuracy using a within-person Goodman–Kruskal gamma correlation as is typically done in the metacognition literature (e.g. Koriat, Ackerman, Lockl, & Schneider, 2009; Son & Metcalfe, 2000). The gamma correlation is the correlation between performance and confidence for participants across trials. Firstly, the CR group had a significantly higher gamma correlation (gamma = .68, 95% CI [.60, .76]) compared with the likelihood group (gamma = .44, 95% CI [.34, .54]). This suggests that the relationship between confidence and performance is stronger in the CR group. While, there are a number of possible interpretations of this finding, it is in keeping with our suggestion that presenting the word ‘confident’ increases the impact of participants’ confidence on performance.

Secondly, we calculated a gamma correlation for each participant individually as a measure of metacognitive accuracy, then examined the interaction between the gamma correlation term and experimental group. Obviously, as the control group did not make task relevant ratings, only the CR and likelihood groups could be compared in this way. The results suggested that the difference between the CR group and the likelihood group did not interact with metacognitive monitoring accuracy (β = −.33, *t* = 1.44, *p* = .155) although given that this result was marginally significant and used a reduced sample a more powerful replication is necessary to rule out the interactive effect between monitoring accuracy and rating type.

### General discussion

The current study examined reactivity to confidence ratings and the extent to which reactivity is moderated by confidence. First, there was no overall reactivity effect for either the CR or prime conditions. However, using a measure of confidence as a moderator (PCA), we showed that high confidence participants tended to experience positive reactivity to confidence ratings (Experiment 1), while low confidence participants tended to show negative reactivity effects (Experiment 2). This is consistent with previous research that establishes the moderating effect of self-confidence on reactivity to confidence ratings (Double & Birney, 2017a, 2017b; Double et al., 2018). In addition, this study was the first to specifically examine the mechanism for reactivity to confidence ratings. We evaluated two distinct hypothesised mechanisms: a priming mechanism, where reactivity is driven by the repeated presentation of the word ‘confident’, and a metacognitive introspection mechanism, which proposed that task-relevant introspection prompted reactivity. Our results provided support for a priming mechanism driving reactivity.

Reactivity to metacognitive ratings has shown inconsistent effects, with some authors observing positive reactivity (e.g. Double & Birney, 2017a; Double et al., 2018; Soderstrom, Clark, Halamish, & Bjork, 2015; Witherby & Tauber, 2017), others observing negative reactivity (Birney et al., 2017; Mitchum, Kelley, & Fox, 2016), and still others finding no reactivity effects (Kelemen & Weaver, 1997; Tauber, Dunlosky, & Rawson, 2015). It has been proposed elsewhere that the direction and magnitude of reactivity is in part determined by task characteristics (Double et al., 2018) or participant characteristics, such a self-confidence (Double & Birney, 2017a). The current findings support such individual differences models of reactivity by showing that self-confidence (measured using PCA) moderates the direction of reactivity to confidence ratings. This is an important finding from a methodological view, because it suggests not only that confidence ratings cannot be considered an innocuous self-report measure when collected during an experiment, but eliciting confidence ratings may exaggerate confidence-related differences in cognitive performance.

The current results suggest that, regardless of whether a rating is task relevant, if the word ‘confident’ is included, then self-confidence-related reactivity is observed. This supports the notion that reactivity is driven by priming participants’ self-confidence, brought about by the repeated presentation of the word ‘confident’. This finding provides an important insight into the nature of reactivity to confidence ratings, in suggesting that reactivity is a specific response to the language of the rating. This provides an obvious recourse to reactivity effects by using more neutral language (i.e. not including the word ‘confident’), which we demonstrated to be somewhat effective in Experiment 2, to the extent that the likelihood ratings group did not show any significant difference from the control group. Therefore, it is advisable that researchers interested in eliciting confidence ratings adopt a more neutral phrasing in order to eliminate unintentional reactivity effects. Furthermore, it suggests that cognitive performance can be enhanced in high self-confidence individuals by priming these self-confidence-related beliefs. This finding is congruent with earlier evidence that suggests that goals and motivation can be unconsciously primed (e.g. Dijksterhuis & Aarts, 2010) and that proximally primed self-confidence can affect performance on an intelligence test (Steele & Aronson, 1995).

Many theories of self-regulated learning espouse the benefits of metacognitive introspection to learning outcomes (Carver & Scheier, 2001; Efklides, 2011). Furthermore, evidence suggests that metacognitive prompts can have a beneficial effect on learning (Bannert, 2006; Bannert, Hildebrand, & Mengelkamp, 2009; Bannert, Sonnenberg, Mengelkamp, & Pieger, 2015). Previous studies that have shown positive reactivity to metacognitive ratings have posited that this may be a result of the metacognitive introspection demanded by such ratings (Double & Birney, 2017a). However, the present findings suggest that there is little benefit to the metacognitive reflection provided by confidence ratings, instead reactivity effects can be found even when task-irrelevant ratings are made, so long as confidence is primed. This suggests that a more controlled approach to the examination of reactivity and the evaluation of metacognitive prompts is needed, as the effects may well be driven by the specific wording of the prompt, rather than the introspection produced, as is often assumed.

In adding to the body of research establishing reactivity to confidence ratings, the current findings have raised methodological issues for the measurement of metacognition. However, the fact that the specific wording of the rating may drive reactivity to confidence ratings provides a clear avenue to reduce reactivity effects by modifying the language used in confidence ratings. In addition, it remains unclear to what extent these priming effects depend on the accuracy of self-confidence judgements. While our results suggest that metacognitive monitoring accuracy (as measured using the gamma correlation) did not interact with the difference between the CR and likelihood groups, the analysis was somewhat limited by the reduced power. Furthermore, the likelihood group cannot be considered a true control group if one wants to examine the effect of monitoring accuracy on reactivity (while reactivity appears reduced in the likelihood group it unlikely to be completely negated). While it is possible that priming the confidence of individuals influences their cognitive performance, regardless of whether they are, in fact, over/under confident in their abilities, this question is deserving of further research.

The present study has provided further support to the notion of reactivity to confidence ratings and replicated the previous findings showing the magnitude and direction of reactivity to confidence ratings is, at least in part, determined by the self-confidence of participants. Furthermore, this was the first study to show evidence that reactivity to confidence ratings occurs due to a priming effect driven by using the word ‘confident’ in the rating. These findings are important for researchers who intend to assess metacognition using confidence ratings and suggest that the use of more neutral language in confidence ratings is an effective way to reduce unintentional reactivity effects.

## Supplementary material

All code and data is available on the Open Science Framework (https://doi.org/10.17605/OSF.IO/F69JA).

## Footnotes

1 As an additional analysis, we examined the quadratic relationship between PCA and performance in both Experiments 1 and 2. In Experiment 1, this effect was significant, *B* = −.27, *p* = .006, but the effect was not significant in Experiment 2, *B* = −.14, *p* = .134. In neither case did the quadratic effect interact with the group effect
